# Preferential Colonization of Metastases by Oncolytic Vaccinia Virus Strain GLV-1h68 in a Human PC-3 Prostate Cancer Model in Nude Mice

**DOI:** 10.1371/journal.pone.0045942

**Published:** 2012-09-25

**Authors:** Ulrike Donat, Stephanie Weibel, Michael Hess, Jochen Stritzker, Barbara Härtl, Julia B. Sturm, Nanhai G. Chen, Ivaylo Gentschev, Aladar A. Szalay

**Affiliations:** 1 Institute of Biochemistry, University of Würzburg, Würzburg, Germany; 2 Genelux Corporation, San Diego Science Center, San Diego, California, United States of America; 3 Department of Radiation Oncology, Rebecca & John Moores Comprehensive Cancer Center, University of California San Diego, San Diego, California, United States of America; 4 Genelux GmbH, Bernried, Germany; 5 Rudolph Virchow Center for Experimental Biomedicine and Institute Molecular Infection Biology, University of Würzburg, Würzburg, Germany; The University of Chicago, United States of America

## Abstract

Recently, we showed that the oncolytic vaccinia virus GLV-1h68 has a significant therapeutic potential in treating lymph node metastases of human PC-3 prostate carcinoma in tumor xenografts. In this study, underlying mechanisms of the virus-mediated metastases reduction were analyzed. Immunohistochemistry demonstrated that virus-treatment resulted in a drastically decrease of blood and lymph vessels, representing essential routes for PC-3 cell migration, in both tumors and metastases. Thus, GLV-1h68 drastically reduced essential routes for the metastatic spread of PC-3 cells. Furthermore, analysis of viral distribution in GLV-1h68-injected tumor-bearing mice by plaque assays, revealed significantly higher virus titers in metastases compared to solid tumors. To elucidate conditions potentially mediating the preferential viral colonization and eradication of metastases, microenvironmental components of uninfected tumors and metastases were compared by microscopic studies. These analyses revealed that PC-3 lymph node metastases showed increased vascular permeability, higher proliferation status of tumor cells as determined by BrdU- and Ki-67 assays and lesser necrosis of PC-3 cells than solid tumors. Moreover, an increased number of immune cells (MHCII^+^/CD68^+^ macrophages, MHCII^+^/CD19^+^ B lymphocytes) combined with an up-regulated expression of pro-inflammatory cytokines was observed in metastases in comparison to primary PC-3 tumors. We propose that these microenvironmental components mediated the metastatic tropism of GLV-1h68. Therefore, vaccinia virus-based oncolytic virotherapy might offer a novel treatment of metastatic prostate carcinomas in humans.

## Introduction

According to current studies, more than 90% of cancer patients die due to direct or indirect effects of metastases. Patient’s prognosis is therefore immediately connected to the metastatic stage of the carcinoma [1]. Metastatic tumor cells infiltrate healthy tissues and cross vessel barriers to access lymphatic or blood circulation. The tendency of a tumor cell to enter lymphatic or blood vessels depends on the ability to adhere to specific structures, such as reticular fibers in the subcapsular sinus of a draining lymph node or endothelial cells that line blood vessels [2].

Prostate cancer is known to metastasize to bones, lungs and lymph nodes [3], [4]. It represents the second leading cause of cancer-related death in men. Since prostate cancer proceeds asymptomatically, the diagnosis is often made when metastases have already formed. Current treatment strategies for metastases are similar to those used for primary tumors [1]. Treatment of advanced prostate carcinoma is performed via conventional chemo- and radiotherapy. Unfortunately, these treatments often result in the development of resistant tumors and metastases [5], [6]. Furthermore, it has been shown that keeping growth of the solid tumor at bay can promote rather than suppress the formation of metastases [7]. Combating both formation and growth of metastases is therefore the key to success in cancer treatment.

Accordingly, oncolytic virotherapy is one of the most promising novel strategies in fighting both: solid tumors and metastases. Oncolytic viruses are able to selectively replicate in cancer cells, resulting in destruction of tumor tissue, but leaving healthy tissues unharmed [8]. In 2007, Zhang *et al.* first described the attenuated recombinant vaccinia virus GLV-1h68 [9], [10]. The oncolytic effect of this virus has been shown in breast, pancreatic and prostate tumor xenografts [10]–[12]. Furthermore, Gentschev *et al.* demonstrated, in principle, the great therapeutic potential of GLV-1h68 in treating lymph node metastases originating from the prostate carcinoma cell line PC-3 [11].

In this study, we characterized the underlying mechanisms of the virus-mediated reduction of metastases. For a detailed analysis, we first visualized metastatic spread of PC-3 cells in the lymph system of nude mice by inserting the *mRFP1*-gene encoding the red fluorescent protein under control of the CMV promoter into the tumor cell genome. Upon viral injection into PC-3-RFP tumor-bearing mice, we demonstrated that virus treatment dramatically decreased the amount of blood and lymph vessels, which are essential routes for metastatic spread of tumor cells, in tumors as well as in metastases [13], [14]. Furthermore, we detected significantly higher virus titers in PC-3 lymph node metastases than in solid tumors and we showed that renal lymph node metastases were colonized to an even higher degree than lumbar ones. To our knowledge, this viral tropism to metastases has, so far, not been described in the literature. Therefore, we set out to analyze mechanisms leading to the preferential viral colonization. In this context, several microenvironmental aspects, such as tumor and metastases vasculature and perfusion, proliferative status and extent of PC-3 cell necrosis, immune cell burden as well as cytokine expression in primary tumors and lymph node metastases were analyzed.

## Materials and Methods

### Cell Lines

The human prostate carcinoma cell line PC-3 (DSMZ ACC465) was cultured in RPMI 1640 (PAA Laboratories, Cölbe, Germany) supplemented with 10% FCS (PAA Laboratories, Cölbe, Germany) and 1% penicillin-streptomycin solution (PAA Laboratories, Cölbe, Germany). PC-3-RFP cells were cultured under same conditions except for adding 10 µg/ml blasticidin. The African green monkey kidney fibroblast cell line CV-1, obtained from the American Type Culture Collection (ATCC CCL-70), was cultured in DMEM High Glucose (PAA Laboratories, Cölbe, Germany) supplemented with 10% FCS and 1% penicillin-streptomycin solution. Human epithelial kidney cells (293FT) were kindly provided by P. Hill (University of Nottingham, originally obtained from Invitrogen) and cultured in RPMI 1640 supplemented with 10% FCS and 2 mM L-glutamine (PAA Laboratories, Cölbe, Germany).

### Generation of PC-3-RFP Cells

The cDNA sequence of the red fluorescent protein (*mRFP1*) was inserted into the PC-3 cell genome using Vira Power™ Lentiviral Expression System Kit (Invitrogen GmbH, Germany) in accordance with the manufacturer’s instructions. The *mRFP1*-encoding plasmid pCR-TK-SEL-mRFP was provided by Q. Zhang (Genelux Corporation, San Diego) and used to generate the *mRFP1-*containing lentiviral vectors as described previously [15]. Replication-incompetent *mRFP1*-encoding lentiviruses were produced in 293FT cells by a co-transfection of the plasmids pLP1, pLP2, pLP/VSVG that supply lentiviral structural and replication proteins and the pLENTI6/V5-DEST-mRFP expression plasmid using Lipofectamine^TM^2000. After transduction of PC-3 cells with mRFP-encoding lentiviruses and blasticidin (10 µg/ml) selection, one stable RFP-expressing PC-3 clone was selected and RFP-expression in >98% of all cells was confirmed by flow cytometry.

### Virus Strain

The attenuated vaccinia virus strain GLV-1h68 was constructed as described previously by Zhang *et al.* [10]. Three expression cassettes encoding for *Renilla* luciferase-GFP fusion protein, β-galactosidase, or β-glucuronidase were recombined into the *F14.5L*, *J2R* and *A56R* loci, respectively, of the parental LIVP virus genome. GLV-1h68 was propagated in CV-1 cells and purified through sucrose gradients.

### Tumor Implantation and Virus Administration

Tumors were generated by implanting 2×10^6^ PC-3 or PC-3-RFP cells in 100 µl PBS subcutaneously into the right abdominal flank of 6–8 weeks old female athymic nude *Foxn1^nu^* mice (Harlan Winkelmann GmbH, Borchen, Germany). A single dose of 1×10^7^ plaque forming units (pfu) GLV-1h68 in 100 µl PBS was injected intravenously (i.v.). For studying the viral colonization of PC-3-RFP metastases, GLV-1h68 was administrated after abdominal lymph node metastases were palpable; usually 45–60 days after PC-3-RFP cell implantation, to mimic the advanced stage of prostate carcinoma. Since the advanced stage of the disease is associated with weight loss independent of viral infection, weight was measured twice a week and mice were sacrificed before standard rates were exceeded. The analyzed treatment periods did not exceed 14 days. After virus injection the health status of the mice did not change.

All animal experiments were approved by the government of Unterfranken, Germany (protocol number AZ 55.2-2531.01-17/08) and/or the Institutional Animal Care and Use Committee (IACUC) of Explora BIOLABS, located in San Diego Science Center (San Diego, USA) (protocol numbers: EB08-003; EB11–25).

### Detection of Human PC-3 Cells in Lymph Nodes via RT-PCR

The presence of human PC-3 cells in enlarged lumbar and renal lymph nodes was analyzed by RT-PCR using primers for human β-actin as described previously [11]. A lymph node was defined to be enlarged when the maximal diameter exceeded 2 mm.

### Fluorescence Imaging of Tumors and Lymph Node Metastases

Images of GLV-1h68 or PBS treated PC-3-RFP tumor-bearing mice were taken either with the Maestro EX Imaging System (Caliper, Hopkinton, MA, USA) or with a MZ16 FA Stereo-Fluorescence Microscope (Leica, Wetzlar, Germany). For imaging of mice with the Maestro EX Imaging System, animals were anesthetized using 2–3% isoflurane. Digital images were processed with Photoshop 7.0 (Adobe Systems, Mountain View, USA).

### Analysis of Viral Titers in Tumors and Metastases

The amount of virus particles in tumor and metastases lysates was determined by standard plaque assays. Therefore, PC-3 tumors and metastases were excised 3, 7 and 14 days after GLV-1h68 injection, minced and 2 volumes of lysis buffer (50 mM Tris-HCl with 2 mM EDTA, pH 7.4) supplemented with 2 mM phenylmethylsulfonyl fluoride and proteinase inhibitor cocktail (Roche Diagnostics GmbH, Penzberg, Germany) were added. Samples were homogenized using a Fastprep Shredder (Thermo Scientific, Karslruhe). After 3 freeze and thaw cycles followed by sonication serial dilutions were titrated on confluent CV-1 cells in 24-well plates. All samples were measured in duplicates.

### Immunohistochemistry

For histology, tumors and metastases were excised and fixed for 16 h in 4% paraformaldehyde/PBS, pH 7.4. Preparation of 100 µm sections and labeling procedures were performed as described previously [16] using the Leica VT1000 Vibratom (Leica, Heerbrugg, Switzerland). After labeling, tissue sections were mounted in Mowiol 4–88 (Sigma-Aldrich, Taufkirchen, Germany). Tissue samples were sectioned (10 µm thickness) with the cryostat 2800 Frigocut (Leica, Wetzlar, Germany). After dehydration in 10% and 30% sucrose (Carl Roth, Karlsruhe, Germany) specimens were embedded in Tissue-Tek® O.C.T. (Sakura Finetek Europe B.V., Alphen aan den Rijn, Netherlands). Cryosections were stored at −80°C and incubated with primary antibodies for 1 h. After washing with PBS, sections were stained for 1 h with secondary antibodies and finally mounted in Mowiol 4–88.

### Antibodies and Reagents

Endothelial blood vessels were stained with a hamster monoclonal anti-CD31 antibody (Chemicon International, Temecula, USA; MAB1398Z) and endothelial lymph vessels with a rabbit polyclonal anti-LYVE-1 antibody (Abcam, Cambridge, UK; ab14917). Non-specific rat-IgG (Jackson Immunoresearch, Pennsylvania, USA; 01200003) was used to analyze the permeability of blood vessels in PC-3-RFP tumors and metastases. Therefore, 10 mg/kg rat-IgG were injected intravenously (i.v.) into PC-3-RFP tumor-bearing mice. Six hours post injection mice were sacrificed and 100 µm sections of tumors and metastases were prepared. Labeling of antigen presenting cells was performed with a monoclonal rat anti-MHC Class II (I-A/I-E) antibody (eBioscience, San Diego, USA; 14–5321). B cells were stained with a rat monoclonal anti-CD19 antibody (Abcam, Cambridge, UK; ab25232), macrophages with a rat monoclonal anti-CD68 antibody (Abcam, Cambridge, UK, ab53444). The proliferation marker Ki-67 was labeled with a rabbit polyclonal anti-Ki-67 antibody (Abcam, Cambridge, UK; ab15580). For analyzing BrdU incorporation into the DNA of PC-3-RFP cells in tumors and metastases, 120 mg/kg BrdU were injected intraperitoneally (i.p.). Three hours later, tumors and metastases were excised and 10 µm sections were prepared. Labeling was performed using rat monoclonal anti-BrdU antibody (Abcam, Cambridge, UK; ab6326) after 30 min incubation with 2 M HCl and washing in PBS. Nuclei were Hoechst 33342-labeled (Sigma Aldrich, Taufkirchen, Germany). DyLight488-, DyLight549- and DyLight 649-conjugated secondary antibodies (donkey) were obtained from Jackson ImmunoResearch (Pennsylvania, USA). All primary and secondary antibodies were diluted 1∶100 in PBS in the case of 10 µm sections or in PBS/0.3% Triton-X-100 in the case of 100 µm sections.

### Fluorescence Microscopy

Microscopic studies were used to compare tumor vasculature, cell proliferation status and specific immune cell populations of tumors as well as of metastases. To examine the fluorescence-labeled tumor and metastasis sections, the following microscopes were used: A stereo-fluorescence microscope MZ16 FA (Leica) equipped with a digital CCD camera and the Leica IM1000 4.0 software (1300×1030 pixel RGB-color images), a TCS SP2 AOBS confocal laser microscope (Leica) equipped with the LCS 2.16 software (1024×1024 pixel RGB-color images) and a Axiovert 200 M microscope (Zeiss) with Axiovision 4.5 software (1388×1040 pixel gray scale images). Digital images were processed with Photoshop 7.0 (Adobe Systems, USA) and merged to yield pseudo-colored pictures.

### Analyses of Digital Fluorescence Images

For analyses of digital images of tumor, LN and RN sections, it is important to note that there was one tumor per mouse, but the number of lumbar and renal lymph node metastases differed from mouse to mouse. In most cases 2 LNs and 2 RNs were present per mouse. Per tumor or metastasis images of 2 sections were analyzed, whereby data obtained from all lumbar lymph node metastases were merged to LN and data obtained from all renal lymph nodes were merged to RN.

#### Measurement of lymph and blood vessel density

Lymph and blood vessel density was measured in digital images (×80 and ×100 magnification) of anti-LYVE-1 and anti-CD31 stained 100 µm sections. Eight images per tumor, LN and RN were analyzed per staining. Exposure time for individual images was adjusted to ensure clear visibility of all detectable blood and lymph vessels and decorated with 8 equidistant horizontal lines using Photoshop 7.0. All lymph or blood vessels crossing these lines were counted to obtain the vessel density per section.

#### Measurement of blood vessel diameters

Blood vessel diameter measurement was performed using digital images (×100 magnification) of 100 µm sections stained with anti-CD31 using Leica IM1000 4.0 software. Images of tumors and lymph node metastases were obtained with individual exposure times to get optimal CD31 signals and exclude signal-dependent variability of vessel diameter. Individual images were overlaid with three equidistant horizontal lines and the diameters of all blood vessels crossing these lines were measured. Blood vessel diameters were determined in 4 images per tumor, LN and RN.

#### Quantification of section labeling

To quantify the permeability of blood vessels, digital images (×160 magnification) of 100 µm histology sections of tumors and metastases were analyzed. The total area positive for IgG (labeled with anti-rat-DyLight488) was determined in 16 different regions per sample. The amount of necrotic tissue in PC-3 tumors or metastases was determined in digital images of 100 µm sections. Nuclei were labeled with Hoechst 33342. The fraction of a section not stained by Hoechst due to nuclei degradation was defined as necrotic area. In this case, images of 2 whole sections per sample were analyzed. The amount of MHC-II, CD19 and CD68 positive cells in PC-3 tumors and metastases was measured in digital images of 10 µm sections. Two whole section images per sample were analyzed. Analyses of the amount of IgG, necrotic tissue and MHC-II-, CD19- and CD68-positive cells in digital images were performed using ImageJ software (http://rsbweb.nih.gov/ij) after converting RGB-images into 8-bit gray scale images using Photoshop 7.0.

#### Measurement of fluorescence intensity

Measurement of the CD31 and Ki-67 intensity was done in digital images of 100 µm and 10 µm sections of PC-3 tumors and metastases. For each staining, 8 different images per sample were acquired with identical settings. RGB-images were converted into 8-bit gray scale with an intensity range from 0–255. The fluorescence intensity of CD31 and Ki-67 stainings represents the average brightness of all staining related pixels and was measured using ImageJ. Images of CD31 staining were taken at 100x, images of Ki-67 at 400x magnification.

### Protein Isolation and Characterization

Protein lysates of PC-3-RFP tumors, lumbar and renal lymph node metastases of 3 mice 49 days after tumor cell implantation were analyzed with an immune-related protein antigen profiling (RodentMAP® v2.0, Rules Based Medicine, Austin, USA) using antibody linked beads. Lysates were performed as described previously [11]. Results were normalized based on total protein concentration.

### Statistical Analysis

A two-tailed Student’s *t* test was used for statistical analysis. *P* values of ≤0.05 were considered statistically significant. Asterisks indicate a significant difference between experimental groups (* indicates p≤0.05; ** indicates p≤0.01; *** indicates p≤0.001).

## Results

### Visualization of the Metastatic Spread of PC-3-RFP Tumor Cells via the Lymphatic System in Nude Mice

**Figure 1:**
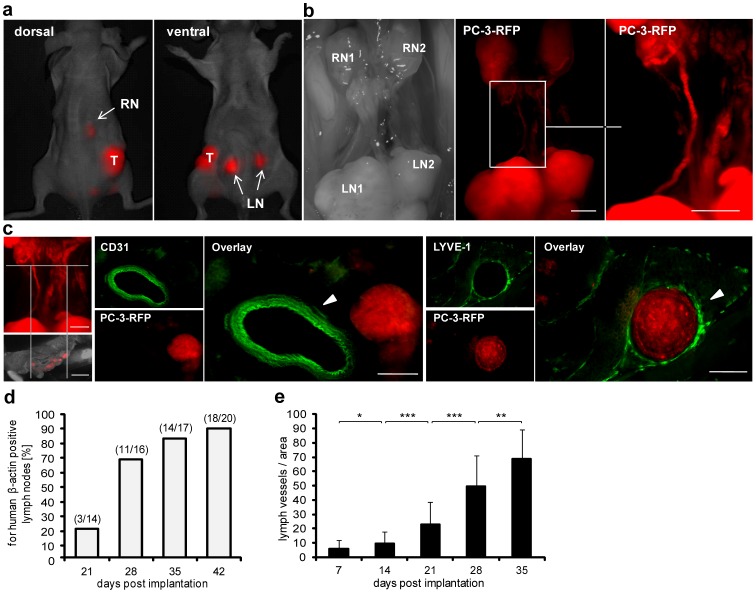
Metastatic spread and correlation between metastases formation and tumor lymph vessel density. 2×10^6^ PC-3 or PC-3-RFP cells were implanted into the right flank of nude mice. (a) Real time imaging of a living nude mouse bearing a PC-3-RFP tumor 70 days post implantation, dorsal and ventral view. T, tumor; LN, lumbar lymph node metastases; RN, renal lymph node metastases. (b) Lumbar (LN1, LN2) and renal (RN1, RN2) lymph node metastases in the abdomen of a PC-3-RFP solid tumor-bearing mouse 65 days post implantation. Right picture: migration of PC-3-RFP cells between LN and RN. (c) 100 µm cross sections of the part between LNs and RNs stained with anti-CD31 and anti-LYVE-1 antibody, respectively. (d) For human β-actin positive lymph nodes 21, 28, 35 and 42 days post implantation of PC-3 cells and (e) density of lymph vessels in corresponding primary PC-3 tumors. Tumors, lumbar and renal lymph nodes of 4 mice were analyzed per time point. To determine the lymph vessel density, 100 µm sections of PC-3 tumors were prepared and 2 sections were stained with anti-LYVE-1 antibody. Four images (x 80 magnification) per section were analyzed as described in material and methods. Scale bars represent 2 mm (b and left picture c) and 200 µm (right pictures c). All images are representative examples.

To analyze the metastatic spread of PC-3 cells within nude mice, it was necessary to establish primary tumors by implanting 2×10^6^ RFP-expressing PC-3 cells subcutaneously into the right flank of nude mice. About 70 days after implantation we visualized metastasized tumor cells in lumbar (LN) and renal (RN) lymph nodes in living PC-3-RFP tumor-bearing mice (Figure 1a). Besides detection in lumbar and renal lymph nodes, PC-3-RFP cells were also visualized in vessels connecting the lymph node pairs (Figure 1b). To figure out whether these vessels are lymphatic or blood vessels immunohistological stainings were performed. Labeling of LYVE-1 (lymphatic marker) and CD31 (blood vessel marker) in tissue sections clearly revealed a lymphatic origin of these tumor cell-containing vessel-like structures (Figure 1c).

Taken together, we visualized the metastatic spread of PC-3-RFP tumor cells from the defined primary tumor site at the right flank to regional lumbar and distant renal lymph nodes in nude mice.

Additionally, a time-based correlation between the formation of lymph node metastases and the density of lymph vessels in the corresponding PC-3 tumors was shown. Over time, a continuous increment of lymph nodes positive for metastasized PC-3 cells was observed (Figure 1d). Simultaneously, lymph vessel density in PC-3 tumors increased (Figure 1e) indicating coherence between the amount of lymph vessels in solid tumors and lymph node metastases formation.

### GLV-1h68 Treatment Dramatically Decreases the Blood and Lymph Vessel Density in PC-3-RFP Tumors and Metastases

Recently, it has been shown that GLV-1h68 treatment of PC-3 tumor-bearing mice results in a significant reduction of lymph node metastases [11]. Additionally, we showed that GLV-1h68 is also an efficient agent in treating PC-3 lung metastases, which mainly arose by metastatic spread of PC-3 cells via the hematogenous route (Figure S1). To understand the therapeutic potential of GLV-1h68, we analyzed the effect of viral treatment on tumor-associated blood and lymph vessels since those play an essential role as routes for metastatic spread to distant organs and lymph nodes [13], [14].

**Figure 2:**
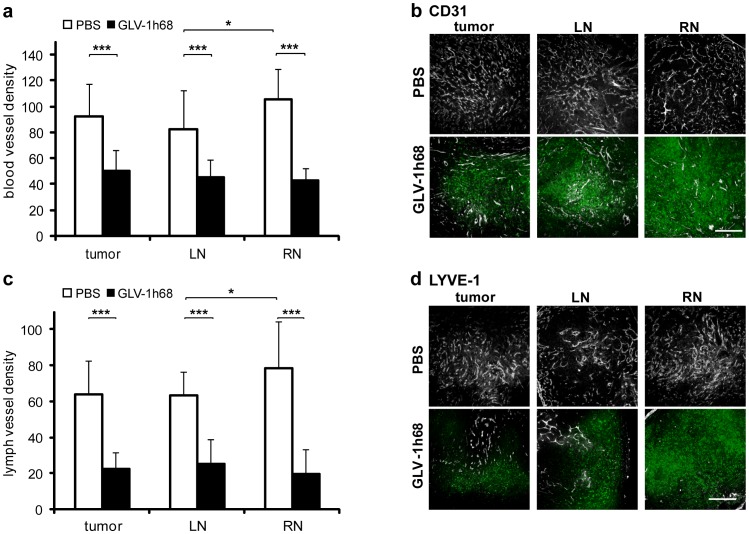
Blood and lymph vessel density in PC-3 tumors and lymph node metastases. Analysis was performed 57 days after implantation of 2×10^6^ PC-3 cells and 7 days after injection of 1×10^7^ pfu GLV-1h68. To determine blood and lymph vessel density, 100 µm sections of tumors, LNs and RNs of 5 PBS- and 5 GLV-1h68-treated mice were stained with anti-CD31 or anti-LYVE-1 antibody. Blood and lymph vessels were counted in 4 images (×100 magnification) from each of 2 sections per sample. (a) Blood vessel density in PC-3 tumors/metastases and (b) representative images of anti-CD31 stained sections. Blood vessels are shown in grayscale, by GLV-1h68 expressed GFP in green. (c) Lymph vessel density in PC-3 tumors/metastases and (d) representative images of anti-LYVE-1 stained sections. Lymph vessels are shown in grayscale, by GLV-1h68 expressed GFP in green. Scale bars represent 500 µm.

For CD31-positive blood as well as for LYVE-1-positive lymph vessels a significant reduction of the vessel density in PC-3 tumors, LNs, and RNs was observed due to intravenous (i.v.) injection of GLV-1h68 (Figure 2). Blood vessel density measurements revealed a reduction of the density by 46% in tumors and LNs and by 60% in RNs in vaccinia virus-injected mice, respectively (Figure 2a and b). Similar results were obtained for the lymph vessel density (Figure 2c and d) indicating that the effect of GLV-1h68 was strongest in renal lymph node metastases.

In summary, the results clearly indicate that oncolytic tumor tissue destruction in PC-3 tumors and metastases is significantly enhanced by eradication of tumor vasculature as well as lymphatic vessels.

### Preferential Colonization of Lymph Node Metastases Compared to Primary PC-3 Tumors by GLV-1h68

**Figure 3:**
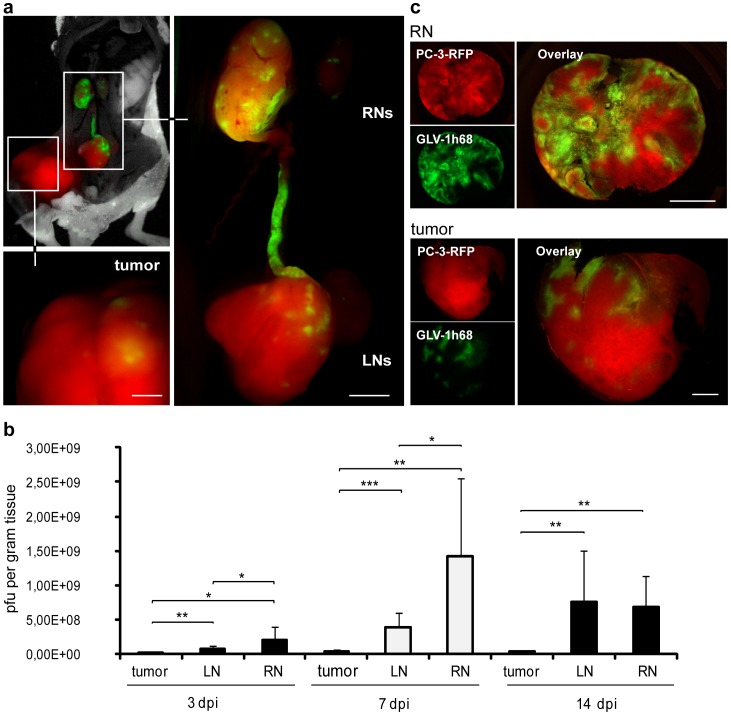
Colonization of lymph node metastases and PC-3-RFP tumors by GLV-1h68. 1×10^7^ pfu GLV-1h68 were i.v. injected into PC-3-RFP tumor-bearing mice. (a) PC-3-RFP tumor-bearing mouse 3 days after injection of GLV-1h68. (b) Virus titers in PC-3-RFP tumors, LNs and RNs 3, 7 and 14 days after injection of GLV-1h68. Virus injection was performed 55 days post tumor cell implantation. Tumors and lymph node metastases of 6 mice were analyzed per group (n = 6). (c) 100 µm sections of a PC-3-RFP tumor and a renal lymph node metastasis 77 days post implantation and 7 days after virus injection. PC-3-RFP cells are shown in red, by GLV-1h68 expressed GFP in green. All images are representative examples. Scale bars represent 1 mm (b) and 2 mm (c).

Since we have observed a stronger reduction of blood and lymph vessel densities in renal lymph node metastases in comparison to primary tumors, we set out to investigate viral colonization patterns of primary tumors, LNs and RNs. To achieve this goal, PC-3-RFP tumor-bearing mice were injected i.v. with 1×10^7^ plaque forming units (pfu) GLV-1h68. To mimic the situation of advanced-stage cancer patients and to ensure the presence of metastases in lymph nodes we injected vaccinia virus in the final stage of the disease 55 days post tumor cell implantation. Multispectral imaging of tumors and metastases revealed clear GFP signals in tumors, LNs and RNs already 3 days post virus injection (dpi). Surprisingly, the GFP intensity was the highest in RNs, followed by LNs and then solid tumors (Figure 3a). Based on these findings we assume a more efficient colonization of lymph node metastases by GLV-1h68 compared to primary PC-3 tumors.

For further analysis, viral colonization patterns for tumors, LNs and RNs were studied in a time course at day 3, 7 and 14 after virus injection by plaque assays. Indeed, it was shown that there were significantly higher viral titers in lymph node metastases compared to primary PC-3-RFP tumors at all three time points (Figure 3b). Interestingly, at 3 and 7 dpi RNs were colonized in a higher degree than LNs. At day 7 post virus injection 1.4×10^9^ pfu of GLV-1h68 per gram tissue were determined in RNs. In contrast, only 28% of RN virus concentration was detected in LNs and as little as 2% in primary tumors. After two weeks, the initial differences in viral titers between LNs and RNs were no longer detectable.

Additionally, microscopic analysis of PC-3-RFP tumor and metastases sections revealed, as expected from higher viral titers, also higher GFP signals in metastases in contrast to solid tumors 7 days post GLV-1h68 injection (Figure 3c). Furthermore, we showed that the preferential colonization of metastases compared to solid tumors by GLV-1h68 is not restricted to lumbar and renal lymph node metastases. Higher GFP signals and viral titers were also observed in inguinal (IN) and sciatic (SN) lymph node metastases (Figure S2). In addition, the investigation of a different route of virus injection revealed similar GLV-1h68 colonization patterns 7 days after intra tumoral (i.t.) injection (Figure S3).

Taken together, these results indicated a highly selective colonization of metastases by GLV-1h68 when compared to primary tumors.

### Analysis of Microenvironmental Factors in Primary Tumors and Metastases Necessary for Enhanced Viral Concentrations in Lymph Node Metastases

To find reasons for the enhanced viral colonization of lymph node metastases, we analyzed the status of PC-3 tumors and metastases before vaccinia virus injection occurred. Various differences in the microenvironment of tumors and metastases that might be crucial for the virus to colonize metastases in a higher degree than tumors had been considered. Therefore, vasculature, proliferative status of PC-3 cells, extent of necrosis, immune cells and cytokine expression in tumors, LNs and RNs were analyzed in uninfected PC-3-RFP tumor-bearing mice. To mimic the situation of advanced-stage cancer patients when diagnosis of cancer usually occurs, we analyzed microenvironmental parameters in the final stage of the disease between 45–60 days post tumor cell implantation.

#### Increased vascular permeability in lymph node metastases

**Figure 4:**
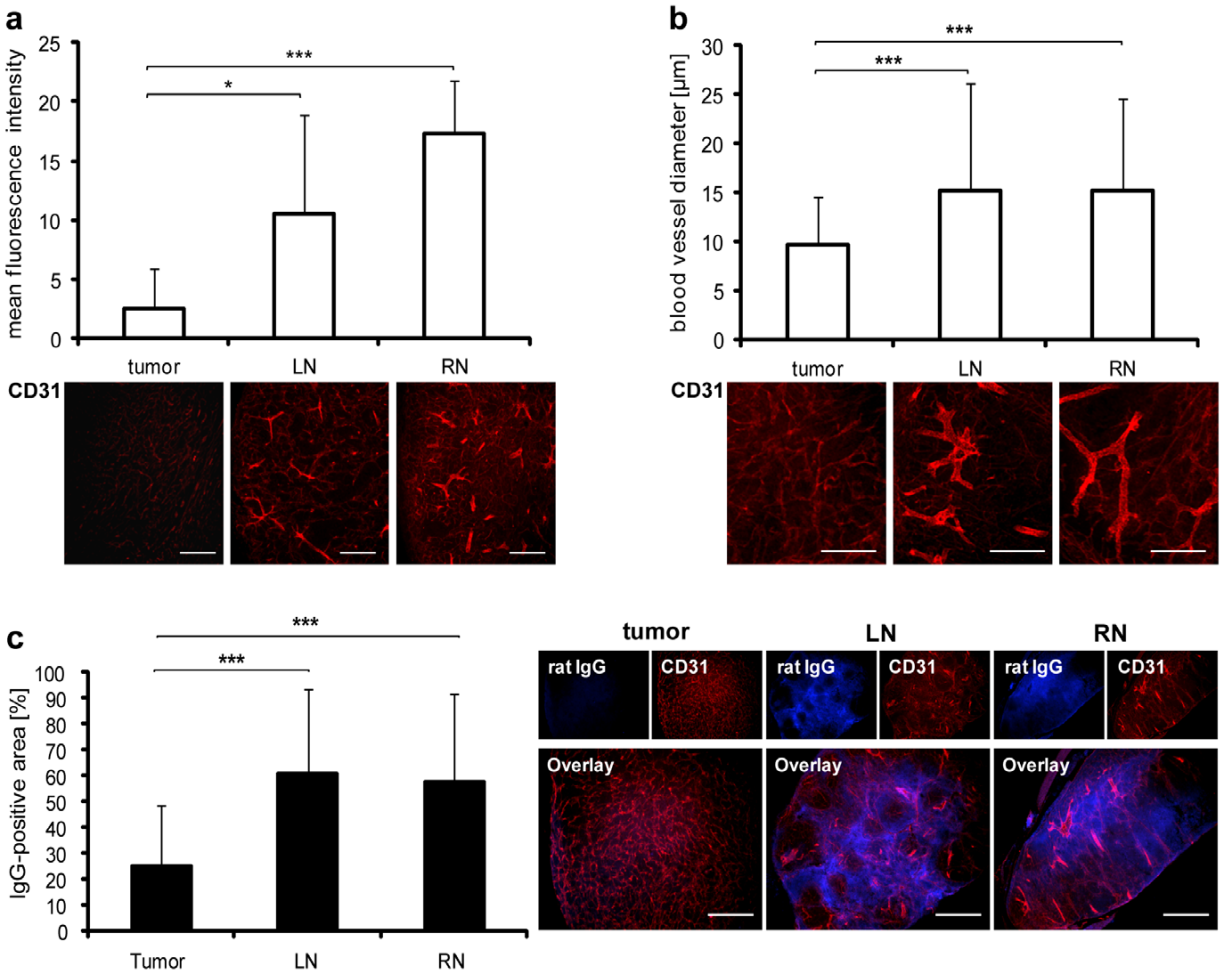
Blood vessels in PC-3-RFP tumors and lymph node metastases. PC-3-tumors, LNs and RNs were excised, cut in 100 µm sections and stained with anti-CD31 antibody. (a) Mean fluorescence intensity of CD31 staining in tumor, LN and RN sections of 5 mice 57 days post tumor cell implantation. The fluorescence signals of 4 images (×100 magnification) from each of 2 sections per sample were measured using ImageJ. (b) Diameter of blood vessels in PC-3 tumors, LNs and RNs of 6 mice 47 days post implantation. Measurement was performed in 2 images (×100 magnification) from each of 2 sections per sample using Leica IM1000 4.0 software. (c) Extravasation of unspecific rat IgGs in PC-3 tumors, LNs and RNs 47 days post implantation. 6 Mice were injected with 10 mg/kg rat IgGs 6 h before tumors, LNs and RNs were excised and histology performed. Extravasated IgG was visualized with Cy2-conjugated anti-rat antibody. Areas positive for IgG were measured in 8 images (×160 magnification) from each of 2 sections per sample using ImageJ. All images are representative examples. Scale bars represent 500 µm (a, c) and 250 µm (b).

First of all, we analyzed whether differences occur in the vasculature regarding density and permeability of vessels between tumors and metastases, leading to a higher initial amount of viral particles within the different tumor tissues. Histology of PC-3-RFP tumors, LNs and RNs was performed and blood vessels were labeled with CD31. Although, no differences in blood vessel density between tumors and metastases were observed 57 days post tumor cell implantation (Figure 2a PBS group), the fluorescence intensity of the CD31 staining was significantly higher in LNs and RNs in comparison to solid tumors (Figure 4a). In general, the blood vessel marker CD31 is highly up-regulated on endothelial cells in inflamed tissues or at sites of ongoing leukocyte transmigration and is associated with higher vascular permeability [17]. Therefore, a detailed microscopic study of blood vessels concerning vessel diameter and vascular permeability in tumors compared to those in metastases was performed. Significantly, LNs and RNs revealed higher blood vessel diameters (mean diameter 15 µm) than solid PC-3-RFP tumors (mean diameter 10 µm) (Figure 4b). To find out whether these dilated blood vessels indeed contribute to higher vascular permeability in lymph node metastases, the extravasation patterns of intravenously injected rat-IgGs were analyzed histologically. Interestingly, significant higher amounts of extravasated IgG were detected in metastases (mean IgG-positive area about 60%) compared to tumors (mean IgG-positive area 25%). However, no significant differences of blood vessel permeability between LNs and RNs were observed (Figure 4c).

These results indicate that the enhanced vascular permeability may result in an increased initial number of viral particles in metastases compared to primary tumors.

#### Metastases exhibited an increased proliferation index and lowered necrosis

**Figure 5:**
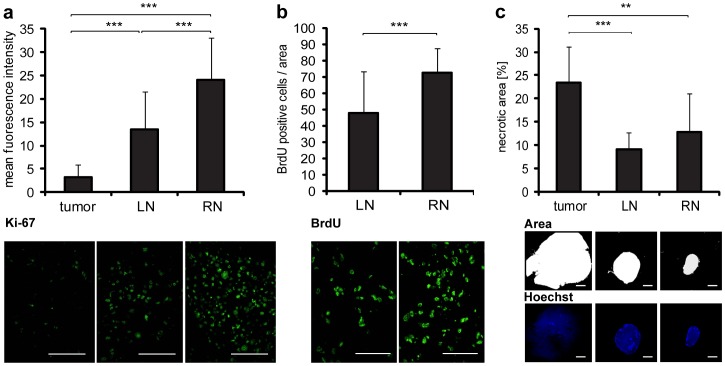
Proliferation status of cells in PC-3 tumors and lymph node metastases. (a, b) PC-3-RFP tumors, LNs and RNs were excised 50 days post tumor cell implantation. 10 µm cryosections were prepared and stained with anti-Ki-67 and anti-BrdU antibodies. (a) Mean fluorescence intensity of Ki-67 staining in tumor, LN and RN sections isolated from 8 mice. The fluorescence signals of 4 images (×400 magnification) from each of 2 sections per sample were measured using ImageJ. (b) Number of BrdU positive cells in LN and RN sections. 8 mice were intraperitoneally injected with 120 mg/kg BrdU 3 h before LNs and RNs were excised. BrdU positive cells were counted in 4 images (×400 magnification) from each of 2 sections per sample. (c) Amount of necrotic tissue in PC-3 tumors and metastases 57 days post implantation. 100 µm sections of tumors, LNs and RNs of 5 mice were stained with Hoechst to label the nuclei. The fluorescence signals in whole section images (×10 magnification) were analyzed. Two sections were measured per sample. The area of a section that was not stained by Hoechst, due to nuclei degradation, was defined as necrotic and measured using ImageJ. All images are representative examples. Scale bars represent 50 µm (a, b) and 2 mm (c).

Since expression of thymidine kinase (TK) positively correlates with the rate of cell proliferation [18] and vaccinia virus replication depends on cellular TK levels [19], the proliferation status of PC-3 cells could directly influence viral replication. For this reason we determined and compared the proliferation index of PC-3-RFP cells in tumors, LNs and RNs by Ki-67 and BrdU assays. Interestingly, we found that the fluorescence intensity of the Ki-67 staining was significantly higher in lymph node metastases than in solid tumors (Figure 5a). Also, marked differences were observed between RNs and LNs, whereby RNs showed distinctly higher Ki-67 fluorescence intensity than LNs.

In addition, we injected BrdU i.p. which ultimately entered the tumors and metastases via the blood vessels. As shown above, there was a significant higher permeability of blood vessels in lymph node metastases compared to the tumor. Therefore, we analyzed and compared only LNs and RNs, due to the general higher perfusion with BrdU in metastases than in tumors. As shown in Figure 5b, a significant higher number of BrdU positive cells was observed in RNs (73+/−15 BrdU-positive cells/area) than in LNs (48+/−26 BrdU-positive cells/area). Furthermore, analysis of necrosis revealed that the area of necrotic tissue was significantly smaller in LNs and RNs than in solid PC-3-RFP tumors (Figure 5c). Approximately 23% of the tumor tissue was necrotic, in contrast to 9% of the LN and 13% of the RN tissue, respectively. However, no significant differences were detected between LNs and RNs.

Thus, a higher proliferation status of PC-3-RFP cells in metastases along with lower levels of necrosis seemed to be favorable for viral replication and dissemination.

#### High density of MHC-II, CD19 and CD68 positive cells in lymph node metastases

**Figure 6:**
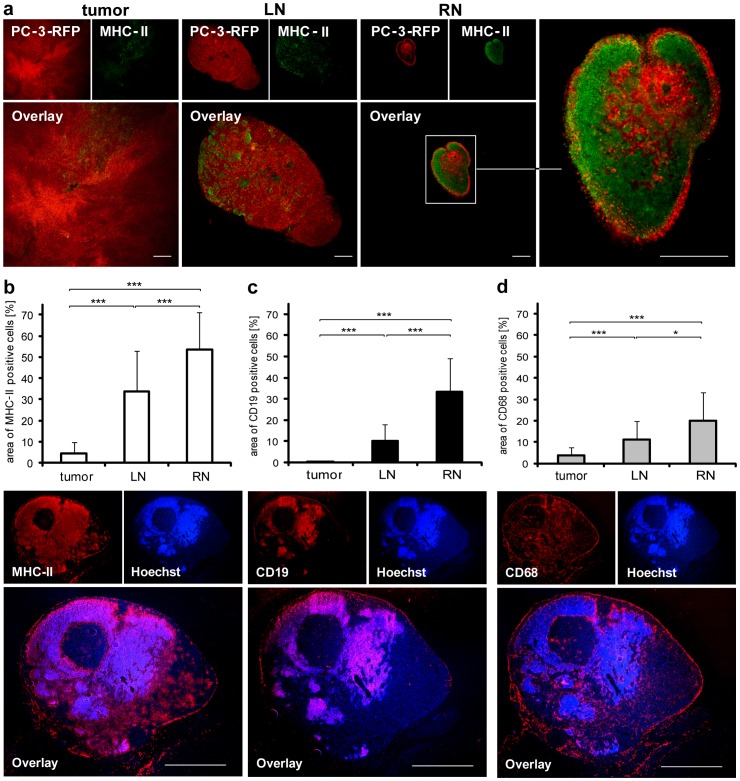
MHC-II-, CD19- and CD68-positive cells in PC-3 tumors and lymph node metastases. (a) Staining of 100 µm PC-3-RFP tumor, LN and RN sections with anti-MHC-II antibody 47 days post tumor cell implantation. (b, c, d) Amount of MHC-II-, CD19- and CD68-positive cells in PC-3-RFP tumors, LNs and RNs. 10 µm cryosections of tumors, LNs and RNs of 8 mice 50 days post implantation were stained with anti-MHC-II, anti-CD19 and anti-CD68 antibody, respectively. Images of whole sections were taken and the area of a section positive for MHC-II, CD19 or CD68 cells was measured using ImageJ. Two sections were analyzed per sample. Below the diagrams are representative images of cryosections of a renal lymph node metastasis stained for MHC-II, CD19 and CD68. Scale bars represent 500 µm (a) and 1 mm (b, c, d).

Lymph nodes represent a target structure for metastasizing prostate cancer cells. Since lymph nodes are essential organs of the immune system where antigen presentation as well as B cell activation and proliferation takes place [20], three antigen presenting immune cell populations were analyzed in lymph node metastases and compared to primary tumors: MHCII- (as monocyte, macrophage, DCs and B cell marker), CD19- (as a B cell marker) and CD68- (as a macrophage marker) positive cells. The MHC-II staining revealed an increased density of antigen presenting cells in RNs compared to LNs and solid tumors (Figure 6a). Furthermore, quantifying MHC-II, CD19 and CD68 stainings resulted in significant increases from tumors to LNs and to RNs in all three cases (Figure 6b, c and d).

In conclusion, we found that the further the distance of a lymph node metastasis from the solid tumor the higher the density of MHC-II-, CD19- and CD68-positive cells in the tissues.

#### Pro-inflammatory signature in lymph node metastases

To analyze the influence of the increased amount of immune cells in metastases, we studied biomarker expression in PC-3-RFP lymph node metastases and in primary tumors of 3 individual mice 49 days after tumor cell implantation using a mouse immune-related protein antigen profiling. Altogether 58 biomarkers were analyzed. We did not observe differences in the cytokine profile of LNs and RNs. Therefore, the data of both were merged und compared to data obtained from primary tumors. The profiling revealed in lymph node metastases a significant (p<0.05) up-regulation of 20 biomarkers, amongst others, a variety of pro-inflammatory cytokines (Table 1) compared to solid tumors. Significant higher levels of chemokines which attract macrophages, neutrophils and dendritic cells, such as IP-10, MDC, MCP-1 and MCP-3 were detected in metastases. Furthermore, chemokines secreted by macrophages, like GCP-2, MIP-1 beta, MIP-2 and MIP-3 beta and growth factors promoting macrophage proliferation, such as GM-CSF and M-CSF-1, were found to be up-regulated in metastases compared to primary tumors. These data indicate that macrophages might play an important role in infection and replication of the vaccinia virus GLV-1h68 in lymph node metastases. Thus, lymph node metastases are characterized by a pro-inflammatory microenvironment, which might promote tumor cell proliferation and virus replication due to increased secretion of cell growth stimulating cytokines.

**Table 1:** Biomarker patterns in PC-3 tumors and lymph node metastases.

Biomarker	Concentration +/− SDin tumors [pg/mg]	Concentration +/− SDin metastases [pg/mg]	p-value	Ratio:metastases/tumor	Classification
Granulocyte Chemotactic Protein-2 Mouse (GCP-2 Mouse)	89.63+/−15.06	564.15+/−240.26	0.0216	6.29	Proinflammatory cytokine
Granulocyte-Macrophage Colony-Stimulating Factor (GM-CSF)	1.23+/−0,38	2.34+/−0,69	0.0086	1.90	Proinflammatory cytokine
Interferon gamma-Induced Protein-10(IP-10)	12.62+/−2.90	17.32+/−8.13	0.0209	1.37	Proinflammatory cytokine
Macrophage Colony-StimulatingFactor-1 (M-CSF-1)	231.57+/−41.87	360.61+/−9.53	0.0205	1.56	Proinflammatory cytokine
Macrophage Inflammatory Protein-1beta (MIP-1 beta)	70.39+/−15.01	108.3+/−145.0	0.0399	1.54	Proinflammatory cytokine
Macrophage Inflammatory Protein-2(MIP-2)	8.58+/−0.633	11.40+/−7.89	0.0351	1.33	Proinflammatory cytokine
Macrophage Inflammatory Protein-3beta (MIP-3 beta)	138.81+/−36.63	1766.5+/−915.7	0.0430	12.73	Proinflammatory cytokine
Macrophage-Derived Chemokine(MDC)	11.27+/−2.651	63.60+/−32.83	0.0177	5.64	Proinflammatory cytokine
Monocyte Chemotactic Protein 1(MCP-1)	61.37+/−13.53	78.43+/−27.53	0.0340	1.28	Proinflammatory cytokine
Monocyte Chemotactic Protein 3(MCP-3)	55.44+/−13.67	67.96+/−39.30	0.0495	1.23	Proinflammatory cytokine

Biomarker expression in PC-3 tumors, LNs and RNs 49 days post tumor cell implantation. Samples of 3 mice were analyzed with a mouse immune-related protein antigen profiling. In the case of significant differences of the biomarker expression in tumors and metastases the ratio (metastases/tumor) was calculated and examples were listed.

Taken together, the presented data strengthened the following tendency: The further the distance of a lymph node metastasis to the solid tumor, the higher the tissue proliferation status and the higher the immune cell burden. Importantly, lymph node metastases showed significantly higher vascular permeability and pro-inflammatory signatures. All of the analyzed microenvironmental parameters positively correlate with increased viral titers and therefore may provide an explanation for the previously observed reduction of metastases in oncolytic vaccinia virus-treated tumor-bearing animals [11].

## Discussion

Metastatic carcinomas represent a major health problem. Therefore, it is crucial to find effective strategies in treating metastases. We focused our study on the formation and microenvironment of lymph node metastases, especially the elucidation of conditions that might lead to efficient colonization with and subsequent eradication of metastases by the oncolytic vaccinia virus strain GLV-1h68.

By inserting the cDNA encoding for *mRFP1* into the PC-3 genome, we generated a simple optical method to visualize metastatic spreading of tumor cells within the vasculature especially in the lymph system of mice. PC-3-RFP cells were detected in lymph nodes as well as in connecting lymph vessels. Although, we were not able to visualize PC-3 cells directly exiting primary tumors and subsequent their migration into lumbar lymph nodes, we hypothesize that this also takes place within lymph vessels. This notion was supported by the observed positive correlation between the lymph vessel density in primary PC-3 tumors and the formation of metastases in lymph nodes.

We described previously that GLV-1h68 showed a significant therapeutic effect on PC-3 lymph node metastases [11]. However, underlying mechanisms of the virus-mediated reduction of metastases are still unknown. Therefore, we set out to investigate microenvironmental parameters contributing to metastases eradication. Interestingly, the analyses of tumor-associated blood and lymph vessels, which are known to be crucial for tumor growth and metastases formation [13], [14], revealed a drastic decrease in the density of both vessel types in PC-3-RFP tumors as well as in metastases upon virus treatment. The decrease of vessel density may eventually lead to a reduction of nutrient and oxygen supply, which in turn may have additive effects on the primary oncolytic tumor tissue destruction by GLV-1h68. Furthermore, migration of PC-3 cells may be limited due to the virus-dependent reduction of essential routes of the metastatic spreading finally leading to fewer metastases. The underlying mechanism of the virus-mediated vessel destruction is so far not resolved. A direct infection of endothelial cells of blood vessels by vaccinia virus leading to vascular collapse is described in the literature [21]. However, we were not able to visualize either infected blood vessels nor infected lymph vessels. It is further suggested that vaccinia virus infection causes massive neutrophil infiltration, resulting in intravascular thrombosis and vessel degradation [22]. We described previously that GLV-1h68 infection induces a strong pro-inflammatory response in tumorous tissues [11], [23]. Furthermore, the strong oncolysis which results in massive tumor cell destruction in tumors and metastases [15] might lead to unfavorable conditions for endothelial cells of blood and lymph vessels, resulting in vessel destruction. In general, methods to inhibit angiogenesis as well as lymphangiogenesis and thereby reducing blood and lymph vessel density are frequently applied to cure cancer and metastases [24], [25]. Data presented in this study suggest that GLV-1h68-treatment simultaneously fulfills both desired approaches in the PC-3 tumor model.

Since the virus-mediated reduction of blood and lymph vessel density was most efficient in renal lymph node metastases, we analyzed colonization patterns of GLV-1h68 in primary PC-3-RFP tumors and in lymph node metastases. Indeed we observed 10–70-fold higher viral titers per gram tissue in lymph node metastases compared to primary tumors at all time points analyzed. These findings might explain both: the stronger reduction of blood and lymph vessel density as well as the stronger oncolytic effect of GLV-1h68 in lymph node metastases. Since analysis was done in the advanced stage of PC-3 carcinoma, it was not possible to analyze time points exceeding 14 days post virus injection. In previous studies from Gentschev *et al*. low titers GLV-1h68 (2.93×10^3^±1.9×10^3^ pfu/gram) have been detected in lymph nodes of PC-3 tumor bearing mice 42 days after virus injection, which means that most of the cancer cells in the lymph nodes have probably been eradicated 6 weeks after virus injection [11].

Taken together, we observed a preferential colonization of metastases by GLV-1h68. Furthermore, based on the data in Figure 3b we found that the larger the distance of a lymph node metastasis from the solid tumor the higher the concentration of GLV-1h68 in the metastasis.

Consequently, we set out to analyze reasons for this metastatic tropism of GLV-1h68. We therefore studied different parameters such as vasculature, proliferative status of PC-3 cells as well as extent of necrosis, immune cell burden and cytokine expression in tumors and metastases before virus injection.

It is reported, that the tumor specifity of vaccinia virus is influenced by the enhanced permeability and retention (EPR) effect, due to size of virus particles [26]. Most solid tumors possess unique pathophysiological characteristics that are not observed in healthy tissues [27]. This includes hypervasculature and greatly increased permeability of blood vessels. To analyze whether there is a stronger EPR effect in metastases than in primary tumors, the vasculature of tumors and metastases was compared. Although no differences of the blood vessel density in tumors and metastases were observed, an up-regulation of CD31 marker protein was detectable in lymph node metastases when compared to solid tumors. CD31 is associated with a higher vascular permeability [17]. Accordingly, CD31 up-regulation in LNs and RNs indicated higher blood vessel permeability in metastases compared to solid tumors, which could alleviate the entrance of GLV-1h68 to metastases. Furthermore, an increased vessel diameter in LNs and RNs compared to solid tumors was observed, which also indicated a higher vascular permeability in metastases [28]. A subsequent extravasation assay with i.v. injected rat-IgG confirmed that the blood vessel permeability is, indeed, significantly increased in lymph node metastases when compared to solid tumors. Therefore, we suggest that a higher EPR effect, mainly characterized by the higher vessel permeability of metastases, may efficiently elevate the initial amount of GLV-1h68 particles in lymph node metastases. It is noteworthy that no major differences in the blood vessel permeability between LNs and RNs were detected. Although the data might explain why lymph node metastases are in general better colonized by GLV-1h68, it does not explain why there are significantly higher virus titers in RNs in comparison to LNs.

To explain this phenomenon, we analyzed the replication status of PC-3 cells in solid tumors and in metastases using Ki-67 and BrdU assays. Due to deletional mutation into the *J2R* gene encoding for thymidine kinase (TK) [10], replication of GLV-1h68 is directly dependent on cellular TK levels [19]. Highly proliferative cells exhibit higher TK levels [18], thus, providing more supportive conditions for virus replication. The Ki-67 assay showed higher amounts of proliferating PC-3-RFP cells in lymph node metastases than in solid tumors. Strikingly, PC-3-RFP cells in RNs proliferate more rapidly than in LNs. This observation was also confirmed by BrdU assays, which revealed significantly higher numbers of PC-3-RFP cells which were positive for incorporated BrdU in RNs than in LNs. In addition, we found that the area of necrotic tissues, which cannot be colonized by vaccinia virus, was significantly smaller in lymph node metastases than in solid tumors. We concluded therefore, that lymph node metastases contain on the one hand PC-3 cells with a higher proliferation status and on the other hand less necrotic regions than the corresponding primary tumors. The higher proliferation rates surely have consequences on the cellular level (such as architecture of the cytoskeleton, availability of nutrients, etc.) which probably facilitate better entry and/or replication of the virus in those cells. Taken together, the observed conditions seemed to be favorable for viral replication and dissemination.

Lymph nodes represent highly proliferative tissues and are essential organs of the immune system where antigen presentation as well as lymphocyte activation and proliferation takes place [20]. In this study, lymph nodes are target tissues for migrating and metastasizing PC-3 cells. Activated immune cells in lymph node metastases may influence viral replication and colonization due to production of cell growth-stimulating cytokines. Significantly higher amounts of MHC-II positive antigen presenting cells, B cells and macrophages were found in lymph node metastases than in solid tumors. Additionally, we observed markedly higher amounts of all three immune cell populations in RNs when compared to LNs. Taken together, we discovered that the further the distance of a lymph node metastasis from the primary tumor, the larger the amount of immune cells and consequently the smaller the amount of metastasizing PC-3 cells (Figure 6a).

By further analyzing the immune cell enrichment in PC-3-RFP metastases, we observed that lymph node metastases exhibit a pro-inflammatory cytokine signature. In fact, significantly higher levels of several pro-inflammatory cytokines were found in LNs and RNs than in the primary tumors. Since most of the cytokines were related to the macrophage population, macrophages, well-known as a source of cytokines which are involved in immune response and inflammation [29], seem to orchestrate the effect of the local metastatic microenvironment more than the B cell population. We showed that growth-stimulating cytokines such as GM-CSF or M-CSF-1 are significantly up-regulated in metastases. These cytokines may be responsible for enhancing tumor cell proliferation which in return helps to accelerate virus replication leading to higher virus titers in metastases. Furthermore, it is conceivable that macrophages and B cells themselves may serve as hosts for vaccinia virus infection facilitating viral spreading and replication within metastases. Sanchez-Puig *et al.* described a high susceptibility of monocytes and B lymphocytes to vaccinia virus infection, these findings support this hypothesis [30]. However, in our laboratories we were not able to observe infected MHC-II positive cells in RNs and LNs upon i.v. administration of GLV-1h68 (Figure S4). Additionally, no vaccinia virus was detected in spleens of tumor-bearing virus-treated mice in previous experiments [10]. The spleen is known to serve as reservoir for monocytes and B cells [31]. Therefore, we propose that these immune cell populations may contribute to virus replication due to cytokine production and establishment of the pro-inflammatory microenvironment, rather than becoming host cells for GLV-1h68 infection. Whether the pro-inflammatory microenvironment will have effects on the adaptive immune system in metastasized lymph nodes in immunocompetent patients cannot be answered at this time as we do not have access to a comparable animal model in immunocompetent hosts. Activation of the adaptive immune system in those metastases could have both negative effects like eradication of the oncolytic virus as well as positive effects such as cross presentation of tumor antigens and therefore even faster eradication of even more tumor cells. Results from current clinical trials (http://clinicaltrials.gov/identifier numbers: NCT01443260 and NCT00794131) will likely answer this question and the effects on metastases should be analyzed in detail in these and similar trials. Even more, since the described pro-inflammatory status is not unique to metastases originating from human prostate carcinoma cell line PC-3, it has also been described in metastatic melanoma cell lines [32]. Therefore, the observed preferential colonization and eradication of metastases by oncolytic rVACVs may not be limited to PC-3 alone.

Taken together, all these findings indicate that the specific milieu of lymph node metastases might contribute to the preferential virus replication in metastases in comparison to primary tumors, finally leading to a more robust oncolytic effect. The elevated vascular permeability in metastases might lead to an increased release of the initial amount of GLV-1h68 particles. Furthermore, lymph node metastases might represent more attractive targets for virus infection compared to primary tumors due to the triggered PC-3 cell proliferation and the lack of necrotic tissues. Finally, the increased numbers of immune cells may also enhance virus replication in metastases by secreting cell growth-stimulating cytokines. The elevated permeability of the vessels of metastases can only enhance the initial viral load immediately after systemic virus injection. Replication differences of VACV in primary tumor cells and metastatic cells are attributable to inherent differences of the respective host cells. Therefore, the different immunological milieu and the proliferation index might be of higher importance for long term viral tumor and metastases colonization. Since metastatic carcinomas represent the major cause of cancer-related death, these findings may become very beneficial to the improvement of the therapy of metastases.

### Conclusion

In this study, treatment of PC-3 tumor-bearing mice with the oncolytic vaccinia virus strain GLV-1h68 led to a distinctly higher infection and destruction of lymph node metastases compared to primary tumors. The virus infection resulted in a large reduction of blood and lymph vessels in tumor as well as metastatic tissues, explaining the recently shown metastases-reducing effect of the virus strain GLV-1h68. Moreover, we demonstrated that non-infected PC-3 lymph node metastases exhibited increased vascular permeability, higher proliferation of tumor cells, lower amounts of necrotic tissues, increased immune cell burden as well as up-regulation of pro-inflammatory cytokines compared to primary tumors. Each of these factors may have contributed to the preferential infection of metastases by vaccinia virus. Since metastatic carcinomas represent the major cause of cancer-related death, these findings may become very beneficial to the improvement of the therapy of metastases. In contrast to the treatment of primary tumors, which can be removed surgically in most cases, fighting metastases faces enormous difficulties, since metastases are often not amenable and surgery is rarely performed [1]. The patient’s prognosis directly depends on the metastatic state of the disease. For this reason, combating metastasis formation represents the key to success in cancer treatment. Thus, combining surgical removal of primary tumors with oncolytic virotherapy using vaccinia virus strains, such as GLV-1h68, leading to an efficient destruction of metastases may become a very promising treatment strategy in fighting advanced prostate as well as other carcinomas in the clinic.

## Supporting Information

Figure S1 **Reduction of PC-3 lung metastases due to GLV-1h68 infection.** Besides in lymph node PC-3-RFP cells could also be detected in lungs of PC-3-RFP tumor-bearing mice indicating the hematogenous route of migration. The RFP fluorescence signal was observed about 60–70 days post tumor cell implantation. Depicted are representative images of a lung 70 days after implantation (a). Furthermore the effect of GLV-1h68 on lung metastases was analyzed. Therefore 5×10^6^ pfu GLV-1h68 was injected i.v. into PC-3 tumor-bearing mice 28 days post tumor cell implantation. At day 21 post virus injection lungs positive for human β-actin, the marker for the presence of PC-3 cells, were determined with an RT-PCR. 5 out of 6 lungs from the mice in the PBS group were tested positive for PC-3 cells. In contrast to this PC-3 cells could not be detected in any of the lungs of mice treated with GLV-1h68 (b). Scale bars represent 100 µm.
(TIF)

Figure S2 **Colonization of inguinal and sciatic lymph node metastases by GLV-1h68.** PC-3-RFP tumor-bearing mice were injected i.v. with 1×10^7^ pfu GLV-1h68. (a) Titers of GLV-1h68 in tumors, inguinal (IN) and sciatic lymph node metastases (SN) per gram tissue 55 days post cell implantation, 14 days after virus injection. (b) Representative images of an inguinal and a sciatic lymph node metastasis 69 days post tumor cell implantation and 7 days after virus injection. Scale bars represent 2 mm.
(TIF)

Figure S3 **Colonization of PC-3 tumors LNs and RNs after i.t. injection of GLV-1h68.** PC-3-RFP tumor-bearing mice were injected i.t. with 1×10^7^ pfu GLV-1h68. (a) Titers of GLV-1h68 in tumors, LNs and RNs per gram tissue 7 days after i.t. (n = 6) and i.v. (n = 6) virus injection. I.v. injection was performed 48 days post cell implantation and i.t. injection 56 days post cell implantation. (b) Representative image of lumbar and renal lymph node metastases 14 days post i.t. virus injection and 64 days post tumor cell implantation. Scale bars represent 2 mm.
(TIF)

Figure S4 **GLV-1h68 infection does not affect MHC-II-positive cells.** Confocal images of RN sections 57 days after PC-3 cell implantation and 7 days after injection of 1×10^7^ pfu GLV-1h68 showed that GLV-1h68 did not infect MHC-II positive cells. All images are representative examples. Scale bars represent 50 µm. Overlay shows GLV-1h68 dependent GFP and MHCII-staining.
(TIF)
