# Supplementary figures and images for: Preferential Colonization of Metastases by Oncolytic Vaccinia Virus Strain GLV-1h68 in a Human PC-3 Prostate Cancer Model in Nude Mice
Source: PLoS One. 2012 Sep 25;7(9):e45942. doi: 10.1371/journal.pone.0045942 (PMC3457966; doi:10.1371/journal.pone.0045942)

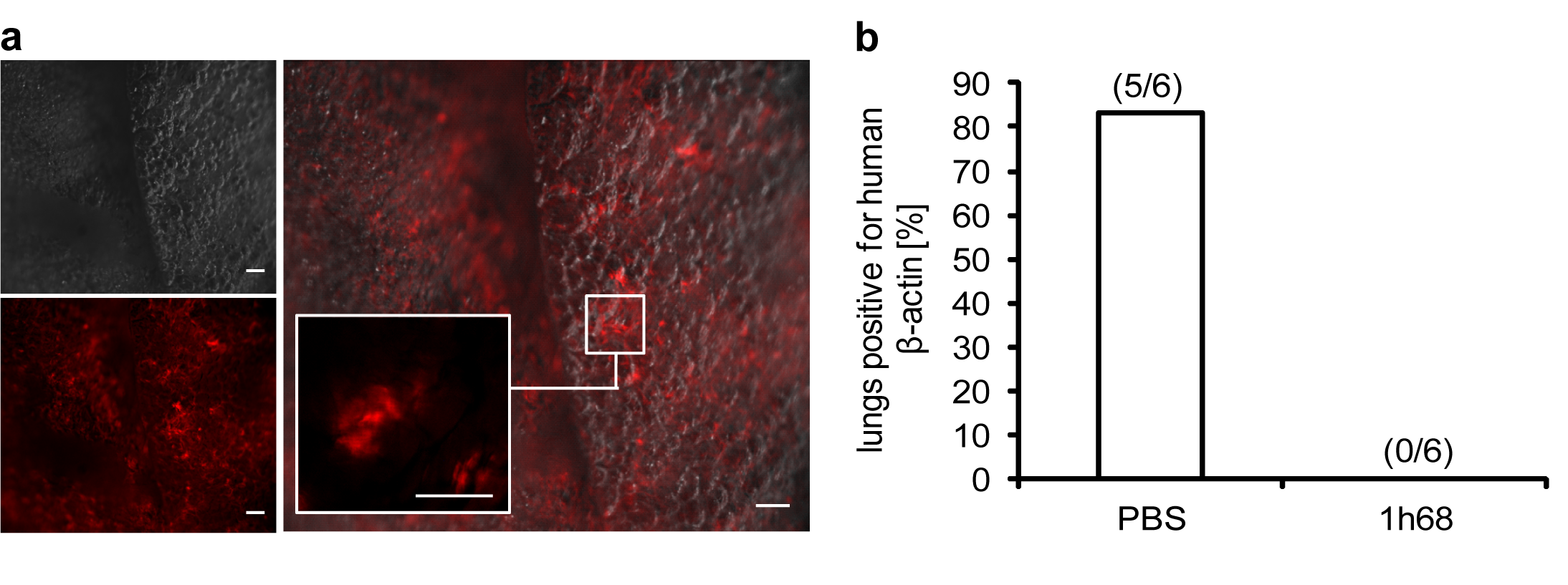

Supplement: Figure S1 — Reduction of PC-3 lung metastases due to GLV-1h68 infection. Besides in lymph node PC-3-RFP cells could also be detected in lungs of PC-3-RFP tumor-bearing mice indicating the hematogenous route of migration. The RFP fluorescence signal was observed about 60–70 days post tumor cell implantation. Depicted are representative images of a lung 70 days after implantation (a). Furthermore the effect of GLV-1h68 on lung metastases was analyzed. Therefore 5×10^6^ pfu GLV-1h68 was injected i.v. into PC-3 tumor-bearing mice 28 days post tumor cell implantation. At day 21 post virus injection lungs positive for human β-actin, the marker for the presence of PC-3 cells, were determined with an RT-PCR. 5 out of 6 lungs from the mice in the PBS group were tested positive for PC-3 cells. In contrast to this PC-3 cells could not be detected in any of the lungs of mice treated with GLV-1h68 (b). Scale bars represent 100 µm. (TIF) [file pone.0045942.s001.tif]

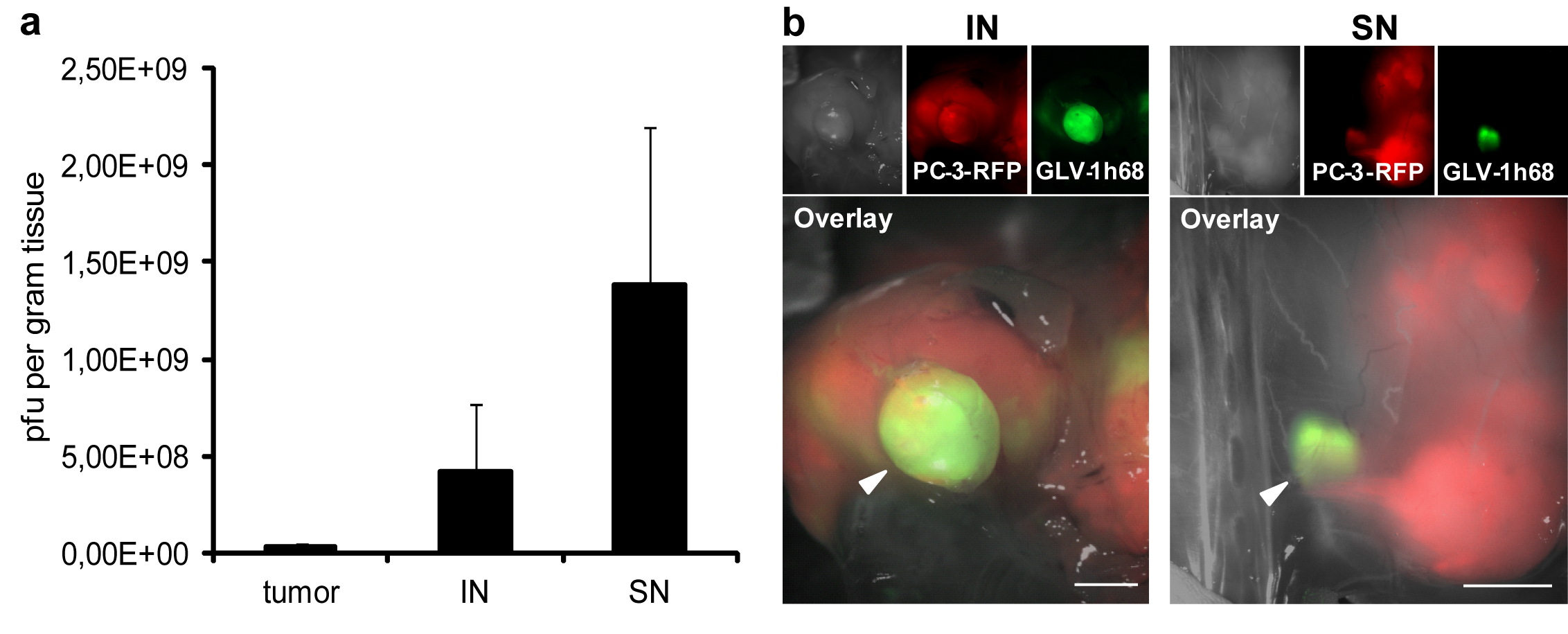

Supplement: Figure S2 — Colonization of inguinal and sciatic lymph node metastases by GLV-1h68. PC-3-RFP tumor-bearing mice were injected i.v. with 1×10^7^ pfu GLV-1h68. (a) Titers of GLV-1h68 in tumors, inguinal (IN) and sciatic lymph node metastases (SN) per gram tissue 55 days post cell implantation, 14 days after virus injection. (b) Representative images of an inguinal and a sciatic lymph node metastasis 69 days post tumor cell implantation and 7 days after virus injection. Scale bars represent 2 mm. (TIF) [file pone.0045942.s002.tif]

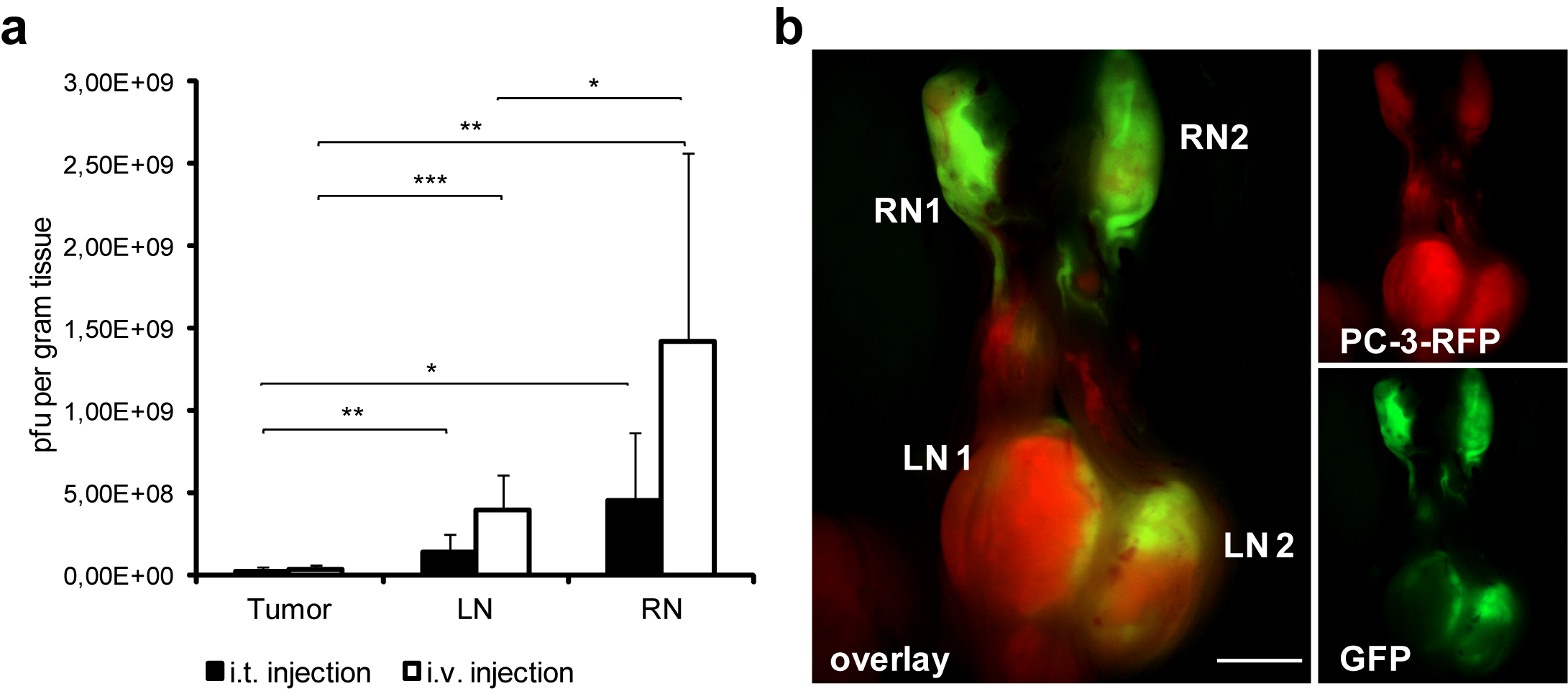

Supplement: Figure S3 — Colonization of PC-3 tumors LNs and RNs after i.t. injection of GLV-1h68. PC-3-RFP tumor-bearing mice were injected i.t. with 1×10^7^ pfu GLV-1h68. (a) Titers of GLV-1h68 in tumors, LNs and RNs per gram tissue 7 days after i.t. (n = 6) and i.v. (n = 6) virus injection. I.v. injection was performed 48 days post cell implantation and i.t. injection 56 days post cell implantation. (b) Representative image of lumbar and renal lymph node metastases 14 days post i.t. virus injection and 64 days post tumor cell implantation. Scale bars represent 2 mm. (TIF) [file pone.0045942.s003.tif]

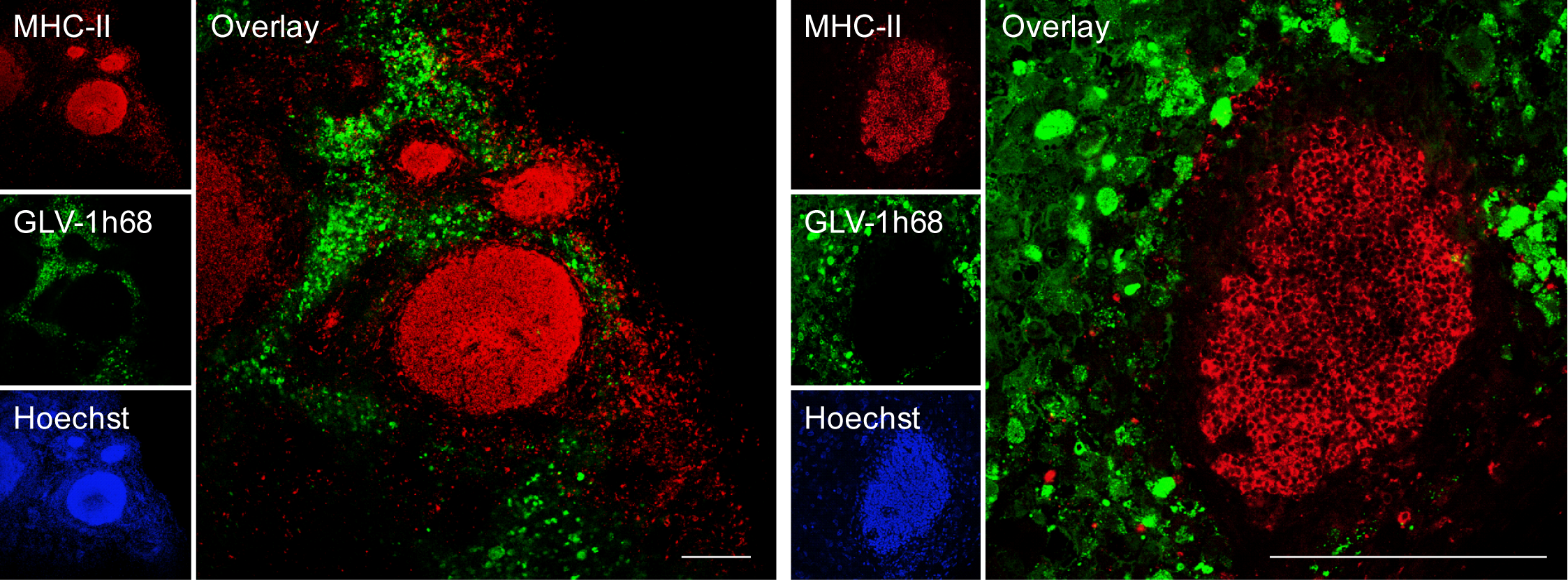

Supplement: Figure S4 — GLV-1h68 infection does not affect MHC-II-positive cells. Confocal images of RN sections 57 days after PC-3 cell implantation and 7 days after injection of 1×10^7^ pfu GLV-1h68 showed that GLV-1h68 did not infect MHC-II positive cells. All images are representative examples. Scale bars represent 50 µm. Overlay shows GLV-1h68 dependent GFP and MHCII-staining. (TIF) [file pone.0045942.s004.tif]
